# Process Safety Considerations in the Design and Scale-Up of Chemical Looping Processes

**DOI:** 10.1021/acs.iecr.5c01678

**Published:** 2025-08-18

**Authors:** Andrew J. Furlong, Nicole K. Bond, Scott Champagne, Jan B. Haelssig, Robert T. Symonds, Robin W. Hughes, Paul R. Amyotte, Michael J. Pegg

**Affiliations:** † CanmetENERGY-Ottawa, 6314 Natural Resources Canada, 1 Haanel Drive, Ottawa, Ontario K1A 1M1, Canada; ‡ Department of Process Engineering and Applied Science, 3688 Dalhousie University, P.O. Box 15000, Halifax, Nova Scotia B3H 4R2, Canada; § Department of Chemical and Biological Engineering, 120472 University of Ottawa, Ottawa, Ontario K1N 6N5, Canada

## Abstract

The technology readiness of chemical looping is rapidly being advanced by transforming batch-mode bench-scale systems into continuously or semicontinuously operating pilot units, and these changes in operating modes and scales introduce new levels of risk. To ensure pilot plants operate in a safe and successful manner and to sustain public support and a positive perception, it is important that a rigorous process safety approach is implemented in both the design and the operations stages of facilities, especially with limited operating history at larger scales. Beyond the application of technically sound engineering of individual unit operations, additional considerations are required for safer operations. The application of the inherently safer design (ISD) principles of minimization, substitution, moderation, and simplification are discussed within the context of chemical looping facilities, and example-based guidance is provided. Particular attention is paid to the selection of oxygen carriers and materials of construction to reduce or eliminate hazards. Passive and active control strategies are briefly discussed for their potential to mitigate accidents in pilot facilities, principally in managing loss of containment through secondary containment, and protecting workers through flame arresting and shielding. Management of change is introduced in a chemical looping pilot plant context, focused on examining alternative configurations and materials, recommissioning plants, and managing documentation and training with high turnover in academic settings. Finally, the need for incident reporting and knowledge sharing related to safety and accidents in the chemical looping community are discussed and recommendations on how this can be implemented are made.

## Graphical abstract

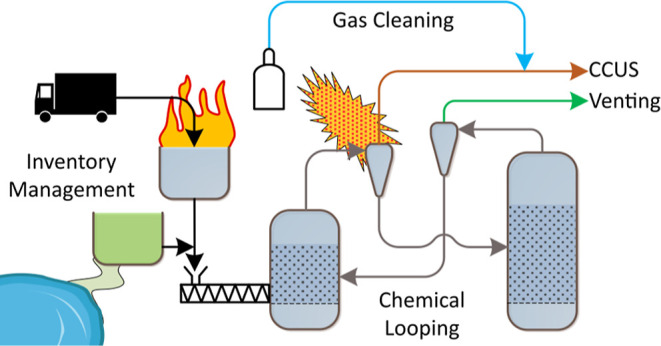

## 1 Introduction

Chemical looping combustion (CLC) and chemical looping reforming (CLR) are promising technologies for the green energy transition, enabling low-cost carbon capture, utilization, or sequestration (CCUS), while continuing to use traditional fuels.^1^ Most CLC processes are implemented as interconnected fluidized beds,^2^ cycling a transition metal/metal oxide between an air reactor (AR) and a fuel reactor (FR), as depicted in Figure 1. The conditions in these processes are extreme, with temperatures up to 1050 °C reported in pilot units,^3^ and pressures up to 5 bar in fluidized bed operations,^4^ with both oxidizing and reducing environments. Higher pressures, up to 95 bar, have been examined for fixed bed CLC at temperatures of 850 °C.^5^ CLC has been examined at scales up to 4 MW_th_ nameplate (up to 5.5 MW_th_ feed),^3^ and CLR at scales up to 140 kW_th_.^6^ Another process design utilizes chemical looping oxygen uncoupling (CLOU), which leverages shifts in metal oxide thermodynamic stability to form gaseous oxygen for reaction with solid fuels.^7^ Synthesis reactions have been proposed for chemical looping; however, these remain at much earlier stages of technological readiness for industrial deployment.

**Figure 1:**
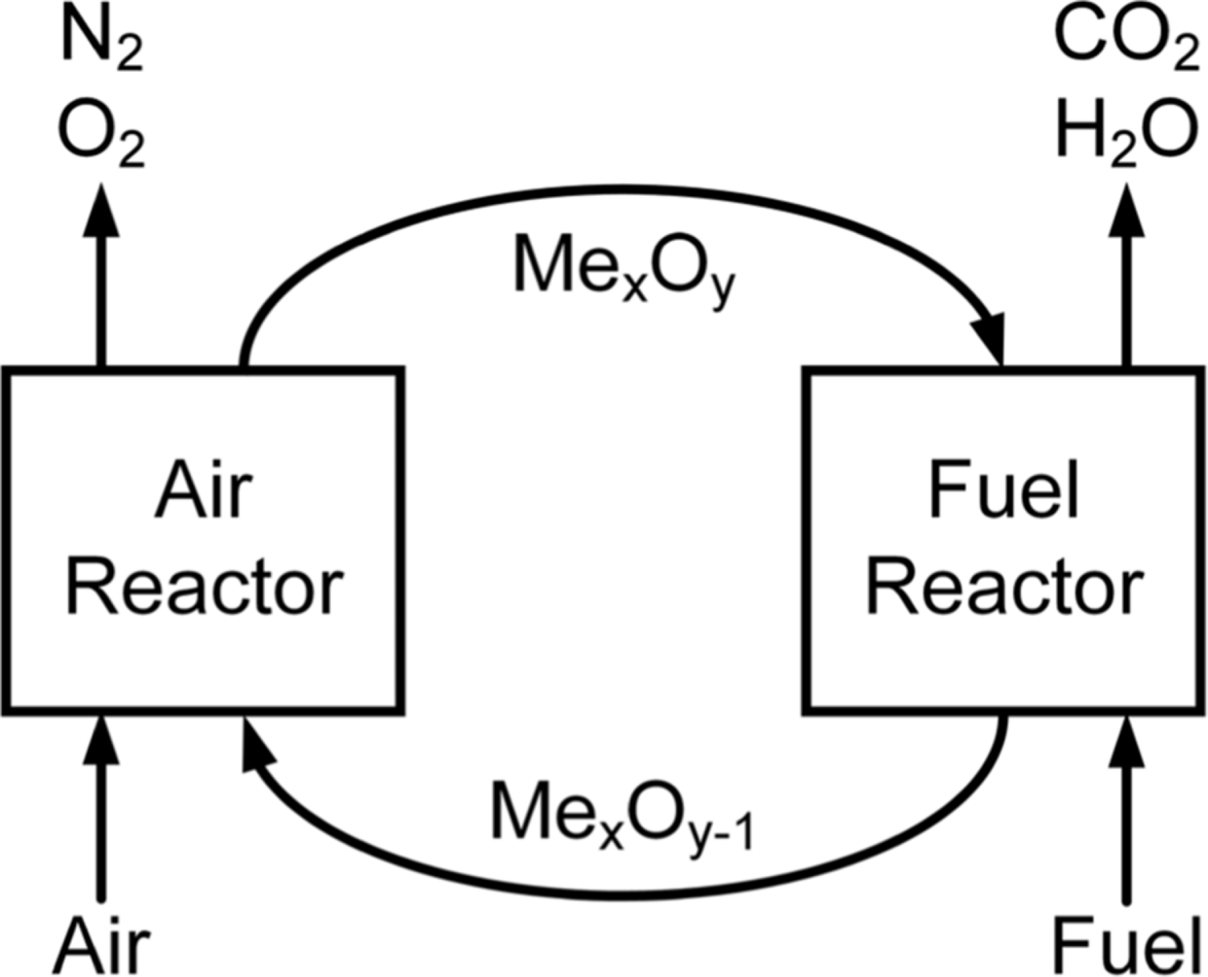
Generalized process configuration for interconnected fluidized bed chemical looping combustion. Reprinted from Furlong et al.^8^ with permission. Copyright His Majesty the King in Right of Canada, as represented by the Minister of Energy and Natural Resources, 2024.

Recently, there has been rapid progress in the scale-up of chemical looping pilot plants (CLPPs). Table 1 summarizes the state of CLPPs with a capacity of at least 100 kW_th_, with the largest CLC plants at 4 MW_th_ nameplate recently having been tested,^3^ and a 3 MW_th_ pressurized plant currently under commissioning.^9^ These pilot plants are several orders of magnitude larger than many of the lab-scale units constructed in the preceding years which provide most operating information,^10^ introducing significantly higher costs and greater consequences in the event of an accident, along with previously unidentified hazards due to changes in process configurations or behavior at larger scales. Further, the new pilot plants are intended for long-term or continuous operations,^3,9^ while previous plants and lab-scale units were operated on shorter timescales of hours to days.^10^ With these increases in costs, operating times, equipment sizes, and number of process units, CLPPs have more hazards and a higher level of risk than previously experienced. However, this transition has not been publicly discussed in the context of process safety, and process safety will play a role in the adoption of any chemical looping technology by industry. With the rapid development of new chemical looping processes and regular new pilot-scale projects, there is the potential for hazards to be unnoticed in the design or operation of a pilot plant.

**Table 1:** Chemical Looping Pilot Plants with a Thermal Capacity of at Least 100 kW_th_

facility (location)	capacity (kW_th_)	reactor design^a^	fuel^b^	first operations
CHEERS (Deyang City, China)^3^	4000	FB	C, P	2023
Alstom (Connecticut, USA)^11^	3000	FB	C	2011
K-CLC (Ulsan, Korea)^9^	3000	FB	M	2024
TU Darmstadt (Darmstadt, Germany)^12^	1000	FB	C	2010
CanmetENERGY-Ottawa (Ottawa, Canada)	600	FB	NG	2025 (anticipated)
KIER (Korea)^4^	500	FB	NG	2019
KIER (Korea)^13^	263	FB	NG	2011
Ohio State University (Alabama, USA)^14,15^	250	MB	S	2017
University of Utah (Salt Lake City, USA)^16^	220	FB	C	2016
SINTEF (Trondheim, Norway)^17,18^	150	FB	B, M	2016
TU Vienna (Vienna, Austria)^19^	140	FB	NG	2008
Chalmers (Gothenburg, Sweden)^20^	100	FB	C, P	2012

a FB: fluidized bed; MB: moving bed.

b B: biomass; C: coal; M: methane; NG: natural gas; P: petroleum coke; S: syngas.

Process safety, as a field, is defined as a means of managing process integrity through design, engineering, and operations, with the aim of reducing loss or harm to people, the environment, processes, or business, through process-related incidents.^21^ Typically, this is considered through the lifecycle of a plant, from the front-end engineering design (FEED) stage through decommissioning, and relies on known information about the process. However, pilot plants are used to develop an understanding of process behavior while handling new materials, in continuous full-scale operating conditions, with unproven system designs and unfamiliar operating conditions.^22^ Because of the wide range of designs and operating modes pilot plants may take, and the novelty of the systems being tested, some hazards may be unknown or overlooked. The importance of safety in pilot plants has recently been highlighted in Chemical Engineering Progress, considering a selection of the general hazards associated with process development for researchers who may be unfamiliar with the topic.^23^

The hierarchy of controls for process safety is a means of ranking process safety measures from most effective to least effective^24^ and is divided into the categories of inherently safer design (ISD), passive engineered controls, active engineered controls, and administrative (procedural) controls. Inherently safer design is generally intended to prevent incidents from occurring but also looks at means of mitigating their effects; however, this relies on process knowledge and planning. Engineered controls, also known as add-on safety, are categorized as either passive or active, with passive controls not relying on any sensing or actuation for performance and thus being deemed safer than active controls, which respond to an event. Engineered safety is generally meant as a means of mitigating effects, although some methods are preventative. These controls can also be added to CLPPs after initial commissioning as process knowledge is developed. A reliance on administrative (procedural) controls, including reliance on personal protective equipment (PPE), should be avoided when possible.

This work discusses important process safety concepts for chemical looping research, especially considering CLPPs are piloting new technologies at larger scales than before. We begin with an overview of hazard identification techniques and apply these to identify many of the unique or hidden hazards present in a CLPP with an extended system boundary including supporting systems. Following the identification of hazards, ISD principles are discussed with example-based guidance for the design, modification, and operation stages of the CLPP. In cases where ISD cannot be employed, passive and active engineered safety systems relevant to chemical looping are highlighted. Management of change is discussed for its role in the operations and recommissioning of CLPPs. We conclude with discussion of the need for knowledge sharing and learning from international experiences regarding incidents among the chemical looping research community to ensure continued success, social license, and growth.

## 2 Hazards in the Chemical Looping Pilot Plant

To ensure a safety analysis is both comprehensive and concise it is important a new boundary be defined for the CLPP beyond the typical research boundary, considering the additional equipment, inventories, and personnel required when operating at large, continuous scales meant to characterize full-scale operations. For the scope of this investigation, the pilot plant boundaries for a general CLPP have been extended to those shown in Figure 2, incorporating supporting piping, vessels, inventories, and rotating equipment. Upstream, this considers the supply of fuel to the reactor (typically a gas or solid, but liquids are possible), air compression, make-up oxygen carrier (OC), and a supply of high-grade oxygen for postcombustion cleanup. Downstream, the process is expanded to include heat recovery, carbon dioxide product purification, product compression for delivery to large carbon dioxide pipelines, and the potential recovery of energy from cooling and expansion. A summary of the identified hazards with potential effects and mitigation is given in Table 2. It should be noted that the analysis presented here is only intended to serve as general guidance as it is not for any specific plant. Quantitative risk assessments (likelihood, severity) are excluded from this work for that reason.

**Figure 2:**
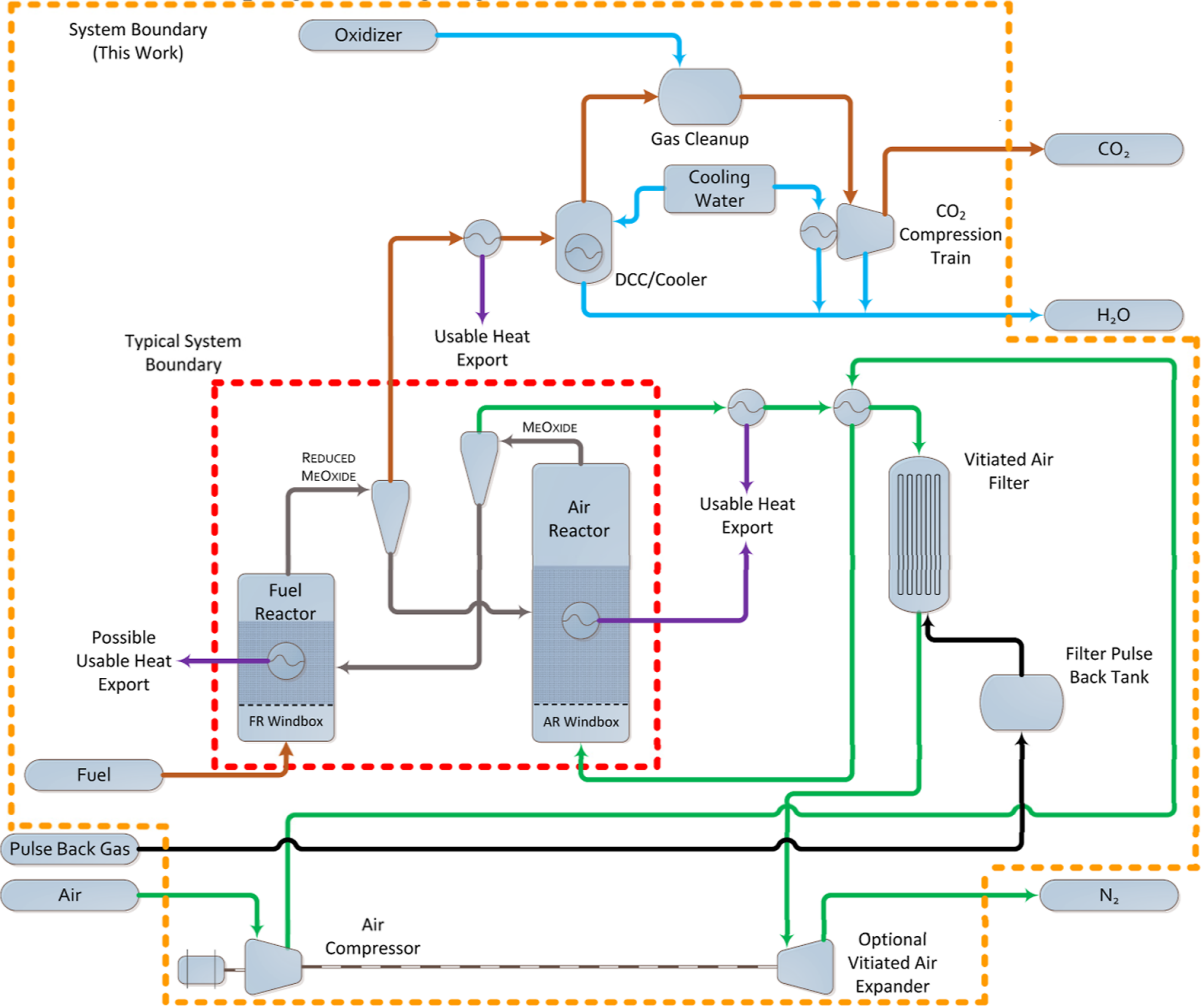
General research-scale chemical looping pilot plant boundaries and expanded scope as considered in this work.

**Table 2:** Summary of Hazards Identified in the Chemical Looping Pilot Plant

hazard	category	sources	possible effects	mitigation
fire	chemical	fuel (gas or solid), OC, product, spontaneous reaction, operational failure, loss of containment	loss of life, process damage, loss of containment	alternative storage, shielding, suppression
explosion	chemical, physical	fuel (gas or solid), OC, product, liquified gases, operational failure	loss of life, process damage, loss of containment, secondary explosions	mechanical overdesign, blast shielding, suppression
oxidizer	chemical	OC synthesis, CLOU, gas polishing	fire, explosion	alternative process configuration
side reaction	chemical	catalysis, materials of construction, poor OC conversion	low-quality/off-spec product, hazardous products, corrosion	materials selection, process monitoring
corrosion	chemical, physical	side reaction, particle-vessel collisions, turbo-machinery erosion, ambient conditions	loss of containment, poor performance/efficiency	process monitoring, materials of construction, manual inspection, solids filtering
loss of primary containment	chemical, physical	secondary event	loss of life, process damage, environmental damage	secondary containment
high temperature	physical	operations	process damage, loss of containment, burns	insulation, process monitoring
high pressure	physical	operations, pressure piling	loss of life, process damage, loss of containment	isolation, pressure relief
human toxicity	health	fuel, OC, product	loss of life	alternative OC, secondary containment, gas flaring
ecological toxicity	health	fuel, OC, product	environmental damage	alternative OC, secondary containment, gas flaring, water treatment
solids plugging	operational	agglomeration, equipment failure	process damage, off-spec product, high pressures	online process monitoring, regular scheduled maintenance, offline inspection
dynamic operations	operational	operations (startup/shutdown), variable feed, system testing	high temperatures, high pressures, off-spec product	control systems, standard procedures
poor knowledge transfer	operational	shift changes, personnel turnover	various	handover procedures, documentation
off-spec product	operational	dynamic operations, variable fuel	business damage	process monitoring, gas flaring

### 2.1 Hazard Identification Methods

Hazard identification and risk assessment are critical in the design and operations stages of any process plant; however, the methods used depend on the knowledge available and the point in the project’s lifecycle. The first methods which can be applied at the conceptual stage are safety reviews and checklist (CL) analyses. In the design stage, other methods, such as what-if analysis (WI), structured what-if analysis (SWIFT), and what-if/checklist (WI/CL) are more general tools that can be used to identify hazard sources. Other methods, used for more detailed engineering, can include the failure modes and effects analysis (FMEA), hazard and operability study (HAZOP), and layers of protection analysis (LOPA). The US Center for Chemical Process Safety (CCPS) has prepared excellent guidance on the use of these tools in the design of pilot plants,^22^ and methods will vary with the experience of the design and commissioning teams.

### 2.2 Previous Hazard Analysis Approaches in Chemical Looping

Discussion of previous approaches taken for safety in the design stages of CLPPs are generally sparse in the literature, as with any industrial design, and publicly disclosed information is focused on technological and economic risk assessment rather than process or operational risks. In process risk analysis, the Chemical ooping Gasification for Sustainable Production of Biofuels (CLARA) project, developing a chemical looping gasification (CLG) process, has conducted a WI analysis, examining several of the core process units.^25^ In recommissioning for gasification operations, the CLARA project has also noted the application of HAZOP.^26^ In the Chinese-European Emission Reducing (CHEERS) project, a combination of hazard identification (HAZID), HAZOP, and LOPA have been employed for CLC and have identified five major potential accident sources; however, the details of these analyses are not public.^27,28^

### 2.3 Chemical Hazards

In chemical process plants, chemical hazards are some of the most obvious, owing to the application of chemical reactions to achieve an end goal or product. In CLPP facilities the primary materials that can be classified as chemicals are the fuel, which can be solid combustible materials such as biomass, coal, or coke, or gaseous fuels, and the OC, which is typically a metal/metal oxide and may be supported by other (typically inert) materials. Other chemicals that can be found in the CLPP include oxygen or other oxidizers stored either compressed or as cryogenic liquids, and product streams such as vitiated air, nitrogen, compressed carbon dioxide, waste streams, and again, combustibles such as hydrogen and carbon monoxide, either pure or in syngas.

The most obvious chemical hazard in combustion-based CLPPs is the flammability of the materials, which can also be extended to their explosibility. While flammability requires the key elements of an ignition source, fuel, and oxidizers, explosions require the addition of confinement and mixing, both of which are present in compact pilot plants, especially those containing fluidized reactors or operating with gaseous or gasified fuels. The explosibility of fuels is well documented for gases such as natural gas,^29^ syngas,^30^ hydrogen,^31^ and carbon monoxide,^32^ with flammability envelopes expanding as temperature increases.^32,33^ Flammable or explosible mixtures can be formed by leaks, failure of piping, loop seal or one-way valve failure, maintenance activities, or as secondary events from other process incidents, both inside and outside of process equipment. Although many CLPPs process solid fuels instead of gases, these can still form mixtures which can create dust explosions. Higher-volatility fuels such as sawdust and woody materials^34^ and lignite^35^ are most likely to be involved in dust explosions. However, some anthracites^36^ and petroleum cokes^37^ have reported explosibility. When handling solid fuels, storage limits must be considered to prevent self-heating or spontaneous combustion.^38^ It has also recently been proposed that OCs may be explosible in the areas surrounding chemical looping reactors including windboxes, ducting, and loading or removal areas, under oxidizing or reducing gases.^8,39^ Explosions involving solids often take place in conveying equipment, but gas–solid separation equipment such as cyclones and in-line filters, and storage silos are also known to have dust explosions occur,^40^ and pose an increased risk with large quantities.

Beyond flammable material, CLPPs may also have oxidizers present. The primary location for this is in postcombustion gas cleanup, where a small supply of air has been used and pure oxygen has been proposed for oxy-polishing.^41^ Oxidizers are also regularly found in OC synthesis operations, such as copper or ferric nitrates as a supply of reactive metal to coat support material,^42^ or nitric acid for leaching when regenerating degraded OCs^43^ as process scales and operating times increase. These operations may be included within site boundaries in future pilot plants.

Additional chemical hazards present include side-reaction products, such as those formed by the catalytic behavior of some OCs. Decomposition of methane to form hydrogen after complete OC reduction has been documented,^44^ however OC materials can also function as reverse water gas shift catalysts,^45^ and nickel-based OCs can function as a steam methane reforming catalyst,^46^ allowing for unintentional production of syngas or its constituents from methane-based sources if conditions allow.

A separate, nonreactive explosion known as a boiling liquid expanding vapor explosion (BLEVE) is also possible in any cases where liquified gases are present. Such gases have been used as or to supplement the oxidizer or fuel,^47^ especially under small, short-term operations where dedicated gas supply lines are not economically viable. BLEVEs can be prompted by external events or by vessel failure, resulting in a loss of pressure and driving phase change. If the fluid is an oxidizer or fuel, this then can create additional reactive events when mixing with the surroundings. BLEVEs have been reported in unloading operations for site use of gases such as oxygen,^48^ and from external sources and overfilling with liquified fuels such as natural gas, propane, or butane.^49^

Extending to chemical hazards with less immediate effects, various forms of corrosion have high potential to cause incidents in larger operations where extended operating times can be expected. Hydrogen embrittlement is a clear concern in any operations processing gaseous hydrogen,^50^ applying primarily to reforming and gasification operations, but also being possible with the previously noted side-reactions. However, high temperature hydrogen attack (HTHA) presents a more serious problem in the CLPP. HTHA affects various steels, especially carbon steel at above-ambient temperatures, and can lead to vessel rupture without apparent warning signs.^51^ In the development of large reactors, carbon steel can appear as an attractive choice when paired with refractory linings, however such linings do not protect the surface, as noted in API Recommended Practice 941.^51^ In particular, refractory degradation, as observed in the CHEERS demonstration unit,^3^ can lead to hot-spots where HTHA is more likely. Degradation of internals may also take place due to sulphuric acid formation with sulfur-containing fuels,^52^ and erosion of internals by particle collisions is also a concern.^53^ For outdoor pilot plants corrosion under insulation is a concern which should be monitored.

The final chemical hazards that should be considered are environmental effects—both to individuals and to the surrounding ecosystem. Many OC materials or byproducts are toxic or are known carcinogens (e.g., NiO, Ni_3_S_2_, NiSO_4_, CoO, CuO),^54,55^ and have been considered for their toxicity if deposited in water systems.^56^ The dust generated by attrition or erosion also poses a hazard to workers during loading and unloading, which is frequently noted on the safety data sheets (SDS) for commercial materials, but for in-house synthesized OCs may not be identified. Finally, there are the toxic or asphyxiant effects of the feed or product gases from chemical looping processes, requiring careful consideration of how exhaust and ventilation should be managed. Working allowances around gases such as carbon monoxide are as low as 25 ppm, while some other species present have allowable time-weighted averages of 1000 ppm or higher, depending on the jurisdiction. Allowable levels are typically included with material SDS.

### 2.4 Process and Operational Hazards

The process conditions in CLPPs contribute to new hazards, due to combinations of extreme temperatures, potentially elevated pressures, and handling of hazardous solids and/or gases. Thermal hazards are especially prevalent in the CLPP and can easily lead to an overpressure event or loss of containment. One such hazard is the potential for changes in the OC material or fuel over a CLPP’s lifecycle, leading to changes in system thermodynamics.^2^ Changing fuels or OCs from a pair with an endothermic reduction reaction to an exothermic reduction reaction may lead to overtemperatures depending on the location of cooling and sensing equipment and sensor delay times, especially in start-up. The increase in scale of systems also poses new hazards, with heat losses scaling nonlinearly with vessel size and nonuniform temperature or concentration profiles, creating hot-spots or areas with poor conversion. The conduction of heat through large, insulated supply lines is also important to consider, with potential for self-heating or devolatilization of solid fuels to occur before the reactor.

Overpressures must also be considered when working with systems involving compression, combustible materials, exothermic reactions, and potential for explosions, whether the system is intended for pressurized operation or not. In pilot plants, consideration should be made for an extended set of circumstances because of the limited information available from bench-scale systems. With the tightly connected nature of CLPPs, there is also potential for pressure piling to occur between vessels, leading to prepressurization of systems and more severe consequences.

Rotating equipment is found in most process plants, for either movement of fluids or for energy recovery. Hazards associated with rotating equipment may relate to high temperatures, variable flow rates, entrained dust, and high failure rates. The primary concern in considering hazards associated with rotating equipment is preventing a loss of containment. In the event of an air compressor failure a loss of circulation and oxidation will occur and may allow fuel to directly pass through the FR. In this case, uncombusted or partially combusted gases can be released, resulting in the previously noted chemical hazards. Pilot plant air compressors may be at higher risk of failure if driven by the expansion of an integrated process stream and require alternative back-up power such as electric drives which can be quickly started. If product compression fails, alternative systems for gas venting may be required to maintain operations without shutdown.

A final operational consideration is the quality of the product, whether this is a hydrogen or syngas stream, or a carbon dioxide product delivered to a pipeline for sequestration or utilization. At present, there are minimal regulations on the quality of carbon dioxide for transport, although many operators specify an upper limit of 2000 ppm carbon monoxide, with some markets as low as 10 ppm.^57^ These limits will also be dependent on the transport method for the carbon dioxide product. The levels of other compounds will vary by operator; however, a baseline including unburnt hydrocarbons (2 mol %), sulphurous compounds (16 ppm total), and oxygen (0.1 mol %), among others, has been used in Canada.^58^ In other lines, an upper limit of 10 ppm oxygen has been given.^57^ Hydrogen purity is dictated by ISO 14687, requiring 99.97% purity and specifying levels of individual contaminants,^59^ with both carbon dioxide and hydrogen lines being designed for dry gas. Process design considering these limits may require additional operations for postreactor treatment, or accommodations for management of off-spec product such as safe product disposal, venting, or flaring.

### 2.5 Dynamic Operations

The intention of any continuous process plant is to maintain stable operating conditions, yielding a desired product, however there are many times where deviation from this operating mode occurs, such as startup and shutdown. Additionally, pilot plants test the boundaries of operation and may be operated closer to or outside of intended limits in order to assess the robustness of systems or may be operated without standard procedures.

The simplest challenge to address in this scale-up is the time requirements for operations, with extended times for startup and shutdown. Extended procedures may cross between operator shifts, and if shift handovers are incomplete or do not capture unexpected operational problems (e.g., failed burners or solids accumulation), referring to standard procedures may return to problems which were previously solved. Knowledge transfer between shifts is also important in the pilot stage when multiple configurations are tested, or the test plan is modified. The second challenge in dynamic operations stages is the integration of CLPPs, where more unit operations are needed for plant and process development, rather than bench-scale research on individual operations. Each operation may need independent control loops, which can be affected by surrounding units. These loops may be especially difficult to develop with the increased system time constants from scaling and the previously noted increase in sensor delay times. Supervisory control systems are necessary at these scales to monitor performance and limit instability or exceedances.

During startup, or restart, there are additional requirements for heating before chemical looping reactions can begin. This energy may be supplied by electrical sources, supplemental fuel, or lances, which require careful monitoring for their performance, ensuring heat is distributed while circulation may not be present. There is also potential for degradation of such sources from erosion or thermal cycling, and extra care should be taken to monitor these on restart. The performance of these heaters can be verified by monitoring electrical consumption or flue gas composition, where appropriate. During standard operations, the same instruments can be used to monitor reactor performance, accounting for variability in natural fuels and, when coupled with operators or control systems, vary the supply of fuel if complete combustion is not achieved.

## 3 Inherently Safer Design

The concept of ISD in chemical process industries stems from Trevor Kletz following the Flixborough disaster in the 1970s, based on the philosophy of “what you don’t have, can’t leak”, and is best applied early in the design of processes to determine how a process can be made safe by design rather than having the severity of consequences reduced by other measures. ISD is generally divided into the categories of minimization, substitution, moderation, and simplification, schematically shown in Figure 3.^24,60^ In the context of this work, the principles discussed and their effects are summarized in Table 3. Some practitioners consider elimination (100% minimization) and isolation to be other means. Equipment isolation, considering the limitation of effects, is typically viewed as a subprinciple of moderation. It is important to note that principles can overlap, especially when evaluating the effects of a design alternative on an overall process.

**Figure 3:**
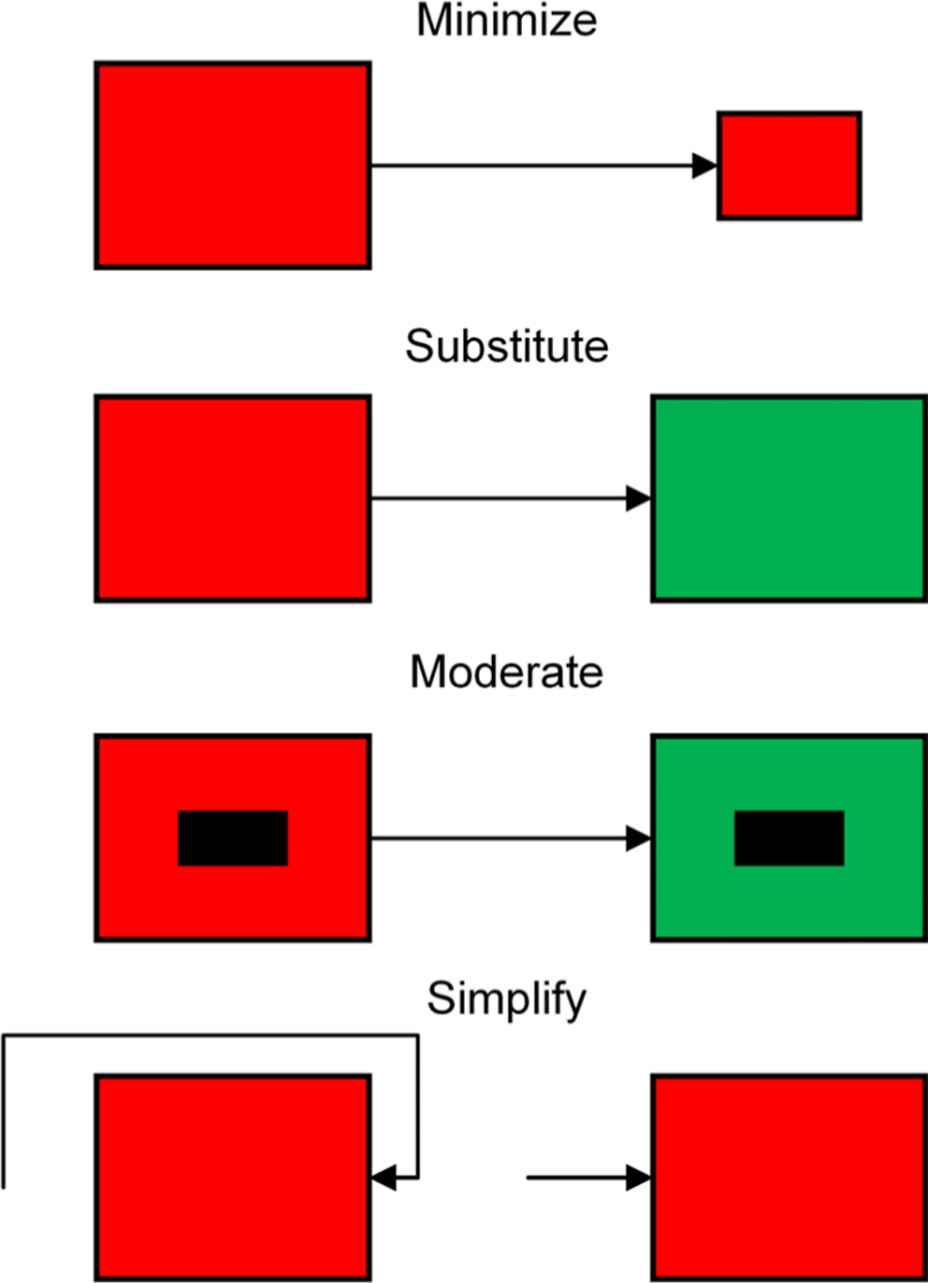
Graphic depiction of the principles of inherently safer design modifying a hazardous process design or chemistry to improve reliability.

**Table 3:** Inherently Safer Design Strategies Discussed in This Work and Their Cascading Effects on Safety

principle in application	ISD principle	contrary ISD principles	primary effects	secondary effects	consequences
fines removal	minimization		controlled reactivity	rotating equipment reliability, reduced fouling	increased operational expense
just-in-time inventory	minimization		reduced fire/explosion/loss of containment severity		potential inventory shortages, turndowns
alternative product gas polishing	substitution		improved product quality		
higher-quality materials of construction	substitution		side reaction and corrosion prevention	improved operational flexibility and plant lifetime	increased capital expense
OC selection	substitution, moderation	minimization	reduced fines production, reduced operating temperatures	reduced temperature swings, ecological protection	limited by fuel and process options, OC quantity may be increased, increased operating expenses
increased pressures	substitution	moderation	reduced vessel size	improved reaction rates, increased latent heat recovery	compression energy requirements, new pressurized vessels
alternative reactor design	substitution, simplification	simplification (process control)	eliminated postreactor cleaning	reduced vessel counts	demanding control and integration
reduced reactor temperatures	moderation	minimization	improved materials reliability		increased reactor size
in-bed heat removal	moderation		reduced system temperatures	improved reactor temperature control	increased heat exchanger degradation
mechanical overdesign	simplification		improved containment in upset conditions	improved operational flexibility	increased capital expense
process automation	simplification		improved upset management		

By its nature, chemical looping is a process modification from fired combustion which implements some principles of ISD, while in its most common forms also implementing some changes contrary to the principles of ISD. Implementing ISD in chemical looping is one way in which safety can be improved in future plant development, and operational reliability and external support can be maintained.

### 3.1 Minimization

In an ISD context, minimization is focused on reducing the quantities of hazardous materials present and reducing the size of process equipment under extreme conditions. The simplest means of implementing minimization in the CLPP is particle size management, both for solid fuels and oxygen carriers, as larger particles will be less reactive in upset scenarios. This is not to suggest that OCs and fuels should be implemented in a larger size than is currently used but rather is focused on removing the smallest particles or fines produced, maintaining reactivity in the desired range. Particle size management can be achieved using additional in-line filtering capturing the smallest solids carried into piping by gases and serves to eliminate damage or fouling to any downstream rotating equipment used for energy recovery. This is especially important for start-up, where high rates of fines production have been noted,^61,62^ and equipment should be designed with variable fines production rates in mind for cleaning and control. Management of fines is also important to evaluate the required replacement rate for OCs. Particle size must also be considered for its effect on total inventories of OC, with potentially increased inventories of material when handling larger particles.

Another approach which is standard for minimization in chemical plants is the use of a just-in-time inventory system for reactive materials,^24^ which can include fuels, OCs and synthesis materials, and oxidizers. Under this approach, hazardous materials are delivered as-needed and kept in small quantities or day tanks, rather than keeping the inventory required for the project on-hand before startup. This approach may be more challenging to implement in short-term pilot plants due to flexible start dates, changing operating plans, and supply chain development, however it can limit the severity of consequences in the event of an incident. In many cases this approach has likely contributed to the absence of accidents.

### 3.2 Substitution

Substitution in ISD is focused on modifying process chemistry to make a process less hazardous. As previously noted, chemical looping modifies traditional fired combustion using a less-hazardous reaction process, removing the use of flames at the expense of larger equipment volumes. Oxy-fuel combustion is a more comparable process to CLC in that the postcombustion separation of carbon dioxide is simplified. However, the use of a solid metal oxide, inert under standard conditions, is inherently safer from an oxidizing hazard standpoint than using pure oxygen. In this case there is potential for additional risk to the environment with the previously noted ecological toxicity of OCs, although this can be partially mitigated with proper containment.

A clear target for substitution in chemical looping is the equipment used for postcombustion gas cleaning, ensuring that products meet regulatory, safety, or sales specifications. As an alternative to in-line oxy-polishing of product gases, near-total conversion can be achieved under less-extreme conditions using catalytic oxy-combustors.^63^ Further applying ISD principles, methods such as absorption, adsorption, and membrane separations can be used to eliminate the use of concentrated oxidizers if some carbon dioxide emissions are deemed acceptable from the process.^64^

The principle of substitution can also be applied to the materials of construction and operation in a pilot plant. Although significantly more expensive than carbon steel, materials such as stainless steel are better suited to operations where hydrogen may be present, either as a reactant, primary product, or side-product, even if refractory linings are used, preventing hydrogen embrittlement or HTHA. Stainless steels may also be better suited than carbon steel to environments where alkali metals are present,^65^ reducing corrosion risks. Using high-alloy steels also allows for more flexibility in future operations. In the selection of the OC, use of mechanically stable materials such as manganese can prevent fines formation, and this has been a major focus for researchers.^66^ Modification to use copper- or manganese-based OCs may also be beneficial for their high conversions of carbon monoxide and hydrogen,^61^ potentially eliminating the need for postcombustion gas cleaning systems entirely.

A final consideration for substitution is the overall design of the process, especially in the design of reactors. Gas switching fixed beds, despite current engineering challenges, can remove the need for postcombustion gas cleaning systems if operated correctly.^67^ Pressurized chemical looping combustion (PCLC), although contrary to the principle of moderation, assists in minimizing the volumes of vessels, which when reviewing the CLPPs identified in Table 1 are large when compared to traditional fired boilers.

### 3.3 Moderation

Moderation in ISD is focused on the use of less-hazardous conditions, such as temperatures, pressures, and concentrations, and in this sense chemical looping is a moderated form of combustion compared to conventional processes such as fluidized bed combustors or standard boilers due to slightly reduced temperatures. Beyond process conditions, moderation can be applied to the materials being used. In the CLPP the use of high temperatures and elevated pressures helps to minimize equipment sizes by increasing reaction rates and conversions, and with the large reactor sizes in demonstration units will be essential for industrial adoption. However, it is important to balance the size of equipment and process conditions when operating at such extreme conditions due to engineering limits. Using copper- or manganese-based OCs is one way in which process conditions can be moderated, with their lower temperatures for similar reactivity,^68^ although these materials are more expensive and prone to agglomeration. It is also imperative to consider the reactivity of the OC with the specific fuels which may be used in this process, considering future changes.

Other means of moderating chemical looping processes can be used outside of the primary reaction vessels, reducing gas temperatures for general CLC and pressures with PCLC immediately upon exiting the reactor. The transport of cooler, low-pressure gases reduces or eliminates many of the hazards identified previously, such as those present with the materials of construction. These reductions can be achieved with fluidized bed heat exchangers as previously discussed by Stenberg et al.^69,70^ in the context of heating other reactions, and being integrated in the K-CLC^9^ and CanmetENERGY-Ottawa reactors under construction and commissioning. Expansion of the gases in PCLC can take place in turbines, assisting the compression of new gas supplies.^71^ Extending beyond the contained process, consideration can be made to locations on-site, with pilot plant operations kept a distance away from fabrication, maintenance, and control rooms, reducing the number of workers and amount of equipment exposed to hazards. API Recommended Practices 752 and 753 provide guidance on this topic.^72,73^

A final means of moderating the process conditions is the use of inert support materials for OCs. The use of inert support materials for their thermodynamic effects on reducing explosion severity has been previously examined,^8^ and with the known attrition-reducing effects of supports^66^ can also be assumed to limit reaction kinetics in upset conditions. It should be noted that the use of a support material will increase the total inventory of OC needed for a reactor, contrary to the principle of minimization.

### 3.4 Simplification

As noted by Hendershot^60^ it may not be possible to eliminate hazards, simply because the property which makes a process hazardous is also the one which makes it useful. In chemical looping the hazards are increased using many interconnected fluidized vessels, as shown in Figure 4, however these vessels are required to separate gases and solids, as well as to keep gas streams separate to maintain product purity for CCUS operations. Applying the ISD principle of simplification, the integration of multiple fluidized vessels as a single element can be used to reduce vessel counts. One such design, shown in Figure 5, is currently under construction at CanmetENERGY-Ottawa for a 600 kW_th_ PCLC reactor. Alternatively, fixed bed chemical looping reactor designs offer some process simplification, removing the need for dedicated gas–solid separations and system balancing while reducing vessel volumes, at the expense of potentially higher reactive vessel counts and increased complexity in gas switching. An alternative design using a rotating reactor bed with internal divisions has been proposed,^74^ removing the challenges created by both fixed beds and fluidized beds, although it has not been investigated with hot flow, and may have additional mechanical complexities.

**Figure 4:**
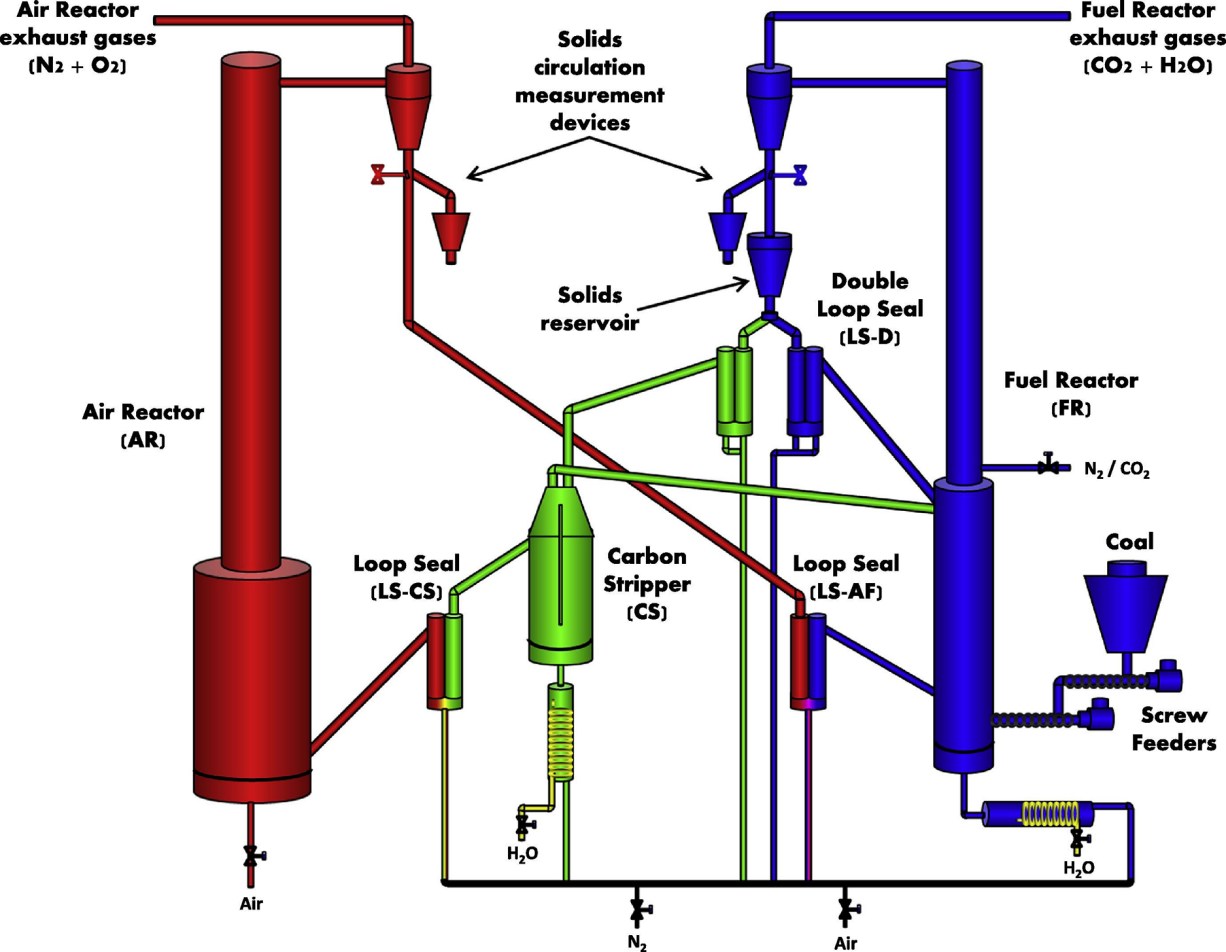
Schematic for the 50 kW_th_ dual fluidized bed CLC unit installed at ICB-CSIC. Reprinted from Abad et al.^75^ with permission. Copyright 2015 Elsevier.

**Figure 5:**
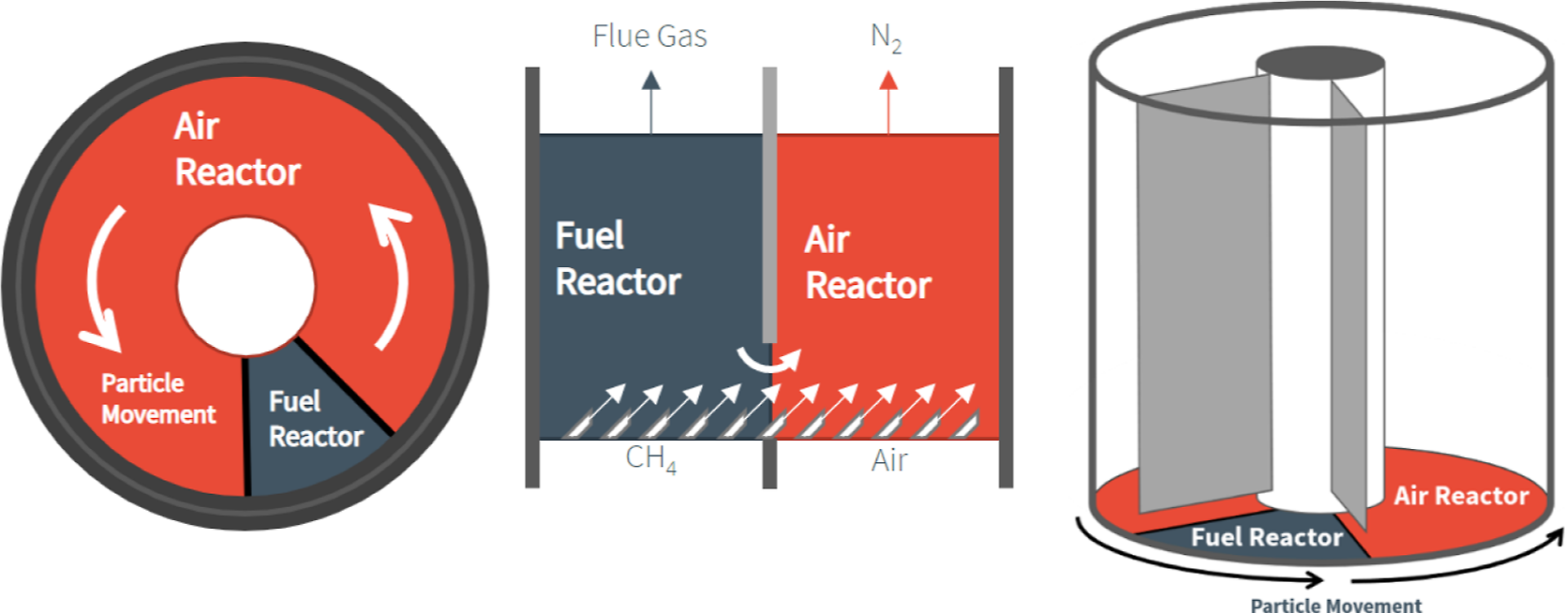
Plug flow internal recirculation reactor concept integrating both air and fuel reactors into a single element.

Although integration of multiple reactor beds into a single unit leads to a simpler design at the plant level, it also introduces additional challenges with process control due to the tight coupling between systems. Pressure balancing and flow control has been a point of discussion in fluidized bed reactors,^2^ and control systems and equipment for gas switching and process smoothing for fixed bed reactors is a major challenge.^76,77^

One other means of simplification which can be applied in the development of pilot plants is designing the equipment, such as vessels, to accommodate upset conditions. In other industrial processes, equipment which is prone to explosions has been designed to handle overpressures,^24^ and the same approach can be taken in the CLPP. If equipment is designed to handle a mild-to-moderate overpressure (more likely than severe overpressures at operating temperatures), the consequences of accidents can be mitigated. This applies to both atmospheric and pressurized equipment, with overpressures in high reactivity events occurring as a temperature-dependent multiplier of initial pressure rather than being additive.

Simplification can also be applied to CLPPs during the operations stage. Simple and clear instructions for operations and management of deviations as they appear in pilot operations are essential for reliability, especially in the event of emergencies. These instructions can be based on extensive hazard analysis using the previously identified methods. Process management in chemical looping may be more complex than other processes, however, and implementation of process control logic may be necessary for simple operator input and reliable process responses.

## 4 Engineered Controls

### 4.1 Passive Engineered Controls

Passive engineered controls can be a simple means of improving plant safety without significant process design changes. Simple measures, such as the incorporation of nonreturn valves, can prevent mixing of reactive gases or liquids and are beneficial especially for systems during startup or shutdown when operations are dynamic. Pressure relief, either through pressure relief valves or rupture discs, is also critical to include in both atmospheric and pressurized units, allowing for venting of gases and/or solids if flow control is lost or systems such as gas filters become plugged. These systems should be examined for their capacity and optimal locations, protecting equipment from unexpected transients, or mitigating explosions if pressure balancing or check valves fail and hot fuel and air mix. Pressure relief installation should be governed by codes, such as the ASME Boiler and Pressure Vessel Code or other regional equivalents. Flame arrestors can also be used to protect against fire or explosion propagation, and some designs exist for flameless venting of solids, such as if there is an explosion in fuel storage.

For improved operator and operational safety, fire walls and blast walls can provide an added level of security to surrounding equipment and operations in the event of fires or explosions. As with facility placement, API Recommend Practices 752 and 753 provide guidance on blast walls for both short-term and long-term operations,^72,73^ both of which may be required in CLPPs. Reviewing the plants in Table 1, CLPP operating lifetimes vary from months to years, demonstrating the need for short- and long-term protection.

For on-site synthesis of large quantities of OC involving liquids, as well as handling liquid fuels, the incorporation of secondary containment dikes provides a temporary measure to prevent environmental damage. With other materials, on-site dump pits can be used as a temporary means of local waterway protection. When developing systems for inventory management in emergencies, consideration should be made for reuse, ensuring systems are still in good working order after each use.

### 4.2 Active Engineering Controls

Active devices in pilot plants can include isolation valves and suppression systems, intended to prevent explosion propagation and pressure piling. Active isolation, in chemical looping, will be important in separating high-risk process vessels like reactors and loop seals from other critical equipment, such as postcombustion units, separations, turbo-machinery, and fuel supplies. These systems can include fast-acting valves, or high rate of discharge (HRD) cylinders, depending on the application.

An additional engineering measure which can be taken to improve safety in pilot plants where CCUS is not implemented is a flare for FR product gases, rather than completing combustion with in-line or catalytic combustion. Flares are typically employed in operations where near-perfect destruction of volatile organic compounds (VOCs), hydrocarbons, carbon monoxide, and hydrogen are required,^78^ which in a pilot plant can be used to manage uncertain process changes and prevent hazardous products from release or accumulation if gases are vented rather than sold. These flares are especially important to meet health and safety or environmental regulations.

Other active systems may be required to improve reactor performance or to account for undesirable operation performance. Systems for clearing lines plugged by solids such as short deliveries of higher-pressure gas can prevent complete shutdown. Similar systems are also required with solids filtering, with pulse back systems used to clear any caked OC. Other systems such as loop seals and surrounding lines may require capability to add more fluidizing gas to periodically improve circulation rates, or account for degradation of gas distributors and reduced performance.

## 5 Management of Change

Management of change (MOC) is perhaps the most critical element to incorporate into the safety management plan for a CLPP, especially if facility life is extended beyond the original scope or operations are restarted. In pilot plants where multiple fuels may be tested, OCs replaced, or configurations altered, engineering review is required to confirm suitability. These reviews will examine the potential for new hazards introduced by changes, consider what other changes may be required, and update documentation such as engineering or construction diagrams and P&IDs to provide reliable information for future development or reconfiguration given the nature of pilot plants. MOC typically will involve technical staff and operators, and in the CLPP may require additional personnel if collaborating internationally to comply with local regulations or standards. Because many operations are also tied to universities, personnel turnover rates are higher than in industrial settings and updating training and technical documentation is necessary for onboarding, but also to note developed standard work practices or operational behavior. Management of organizational change is thus a critical component of overall MOC efforts.

## 6 Knowledge Sharing

A review of the literature on chemical looping shows no publicly reported major incidents with detailed investigation. However, as is regularly seen in other industries, many serious incidents are unreported, underreported, or ignored,^79^ and chemical looping may be no different. Although the design of every CLPP is different, and management falls under different research and industry groups, plants all share similarity in their chemistry, operations, and procedures which can benefit from new knowledge. Most importantly from a research perspective is that these facilities all aim to demonstrate technology viability at commercial scales, with public perception and support for future development depending on the continued success of other operations. In order to maintain this support, it is imperative that repeated occurrences of the same process incidents do not occur, which can be facilitated by operators reviewing their incidents internally to identify root causes in safety management, and immediate or technical causes. After causes have been identified, dissemination of findings to other current or prospective operators is critical to ensure other facilities are not susceptible to the same problems, and that operations can proceed in a safe and loss-free manner.

In the United States, the US Chemical Safety and Hazard Investigation Board (CSB) has investigated numerous incidents, in collaboration with operators and safety experts, providing technical and root cause analyses, and issuing public reports on the findings and recommendations to individual operators and regulators.^80^ Similar content is produced by ARIA in France,^81^ and incident statistics and descriptions are recorded in the eMARS system hosted by the European Commission on the MINERVA Portal.^82^ Case histories are also included in the Loss Prevention Bulletin published by IChemE for a general process engineering audience,^83^ and other histories compiled in books.^84,85^ These reports are well-received and provide excellent guidance to prevent recurrence of incidents at other sites, ultimately boosting perceptions of having chemical process plants in an area. General guidance for plant operators has been provided by other newsletters, such as the ICI safety newsletters produced by Trevor Kletz and later Alan Rimmer^86^ and the Process Safety Beacon published by the CCPS.^87^ In specific industries, groups such as Dust Safety Science^88^ have formed, providing more targeted information to readers. Such content can be shared on a regular basis in newsletter format, including information from past incidents as a reminder or a lesson for new operators, as well as recent histories. A summary newsletter, produced in a rotation of research groups and providing nontechnical content is one form which this could take. Alternatively, regular discussion at field-specific conferences (e.g., International Conferences on Chemical Looping) where key members of research groups will meet can be used to inform other operators of safety concerns. It should be noted, though, that this will likely provide information in a less timely manner and may be missed.

## 7 Conclusions

Chemical looping pilot plants offer a means of scaling transition energy technologies from short-term bench experiments operated in university laboratories to megawatt-scale industrial operations used for continuous heat, power, or chemical production. While this shift in scale is necessary to progress chemical looping as a research and industrial field, it also introduces new hazards and new levels of risk. This work has identified many of the hazards that can appear in an expanded system boundary considering the extra equipment required to meet legal or social requirements for a pilot-scale facility and has provided guidance on management of these hazards through inherently safer design, engineered controls, and management of change. Key hazards identified are associated with unintended reactions, with side-reactions via catalysis and materials of construction especially noted. Hazards associated with maintaining inventories of fuels, oxygen carriers, and materials for start-up and gas cleaning are also considered. Operational hazards from dynamic stages and potential overpressure sources are also noted.

Inherently safer design can be applied to many aspects of chemical looping pilot facilities and has the potential to prevent—rather than mitigate—any disastrous consequences. Many of the strategies noted here are focused on oxygen carrier management, from conceptual stages with materials selection to management of inventories and recycling. Continued development of oxygen carriers can be used to eliminate some hazards and can assist in removing additional downstream operations. Using stainless steels for pilot-scale operations is recommended to improve flexibility and safety when considering the noted hazards, especially high-temperature hydrogen attack and high operating temperatures. The modification of processes, both at the reactor and overall plant level, also offers opportunities to improve reliability. Process simplification through modified vessel arrangements are noted for their potential to decouple systems or eliminate hazards entirely. These methods, however, are generally unproven in pilot scale looping reactors.

Engineered controls beyond standard design practices can be used to mitigate the effects of process accidents and can be implemented with relatively minor process changes. Management of fires and explosions by isolation, both internal to the process and externally, can help in mitigating consequences. Gas and liquid release management through secondary containment and flaring offer means of limiting harmful effects in the event of spills, or when not sending product to a consumer during test stages.

Management of change has been identified as a key point to consider in the lifecycle of the pilot plant, especially when testing alternative process configurations or when restarting facilities with new materials after shutdowns. Finally, the importance of knowledge sharing has been discussed, with the success of groups such as the US CSB, ARIA, and Dust Safety Science highlighted, and the need for incident reporting and investigation even within the research and pilot plant community noted. Sharing between research laboratories through electronic newsletters and conferences is proposed as a solution.
